# The Mingle-Mangle of Wnt Signaling and Extracellular Vesicles: Functional Implications for Heart Research

**DOI:** 10.3389/fcvm.2018.00010

**Published:** 2018-02-22

**Authors:** Julia Christina Gross, Laura Cecilia Zelarayán

**Affiliations:** ^1^Hematology and Oncology, University Medical Center Göttingen, Göttingen, Germany; ^2^Developmental Biochemistry, University Medical Center Göttingen, Göttingen, Germany; ^3^Institute of Pharmacology and Toxicology, University Medical Center Göttingen, Göttingen, Germany; ^4^Partner Site Göttingen, German Centre for Cardiovascular Research (DZHK), Göttingen, Germany

**Keywords:** extracellular vesicles, exosomes, Wnt signaling, Wnt secretion, heart remodeling

## Abstract

Wnt signaling is an important pathway in health and disease and a key regulator of stem cell maintenance, differentiation, and proliferation. During heart development, Wnt signaling controls specification, proliferation and differentiation of cardiovascular cells. In this regard, the role of activated Wnt signaling in cardiogenesis is well defined. However, the knowledge about signaling transmission has been challenged. Recently, the packaging of hydrophobic Wnt proteins on extracellular vesicles (EVs) has emerged as a mechanism to facilitate their extracellular spreading and their functioning as morphogens. EVs spread systemically and therefore can have pleiotropic effects on very different cell types. They are heavily studied in tumor biology where they affect tumor growth and vascularization and can serve as biomarkers in liquid biopsies. In this review we will highlight recent discoveries of factors involved in the release of Wnts on EVs and its potential implications in the communication between physiological and pathological heart cells.

## Wnt Signaling Pathways Overview

Wnts are evolutionarily conserved, secreted glycosylated growth factors, which in humans are encoded by 19 different Wnt genes. There are more than 15 different Wnt receptors and co-receptors, including Frizzled (FZD1-10), LRP5 and 6, and ROR1/2 that are best described. Depending on their binding to receptors and downstream components, Wnt signaling has been classified into canonical (β-catenin-dependent) or non-canonical (β-catenin-independent) pathways. The β-catenin-independent pathways include Planar Cell Polarity (PCP) and Wnt–Ca^2+^ pathway [reviewed in (1)].

The β-catenin-dependent pathway is activated by binding of Wnts with FZDs and LRP5/6, and subsequent GSK3β inhibition, leading to stabilization of cytoplasmic β-catenin. Upon accumulation, β-catenin enters the nucleus binds to TCF (T cell factor)/LEF (lymphoid enhancer-binding factor) transcription factors and regulates the transcription of target genes. Additionally, β-catenin-independent Wnt pathways use different downstream signaling modules. The PCP signaling is activated via FZDs receptors with ROR1/2 and PTK7 as co-receptors, through a cascade of small GTPases RAC1, RHOA and JUN-N-terminal kinase (JNK) activation. This pathway leads to changes in cytoskeleton, cell polarity and activation of JNK-dependent transcription factors and their target genes [reviewed in (2)].

A second β-catenin-independent pathway is the Wnt–Ca^2+^pathway. Here, Wnts trigger FZD-mediated activation of heterotrimeric G proteins. This activates phospholipase C (PLC), diacyl-glycerol (DAG) and inositol-1,4,5-trisphosphate [Ins(1,4,5)P3] cascade triggering intracellular Ca^2+^-release and activation of effectors such as calmodulin-dependent kinase II (CAMKII), calcineurin and protein kinase C (PKC), which activate the transcriptional regulator nuclear factor associated with T cells (NFAT) (3).

In addition to Wnts, several inhibitors, such as Dickkopfs (DKK1-3), secreted Frizzled related proteins (SFRP1-5) and activators, such as R-Spondins (RSPO1-5) are similarly secreted and can regulate the amplitude and specificity of Wnt signaling at the extracellular level (4). Additionally, intracellular cytosolic and nuclear inhibitors fine-tune Wnt signaling.

## Wnt Component Mobilization in Extracellular Vesicles

Currently, increasing evidences show the importance of different Wnt signaling branches and their crosstalk among different cell types. Secreted Wnts are lipidated and this hydrophobic moiety hinders them to freely move in the extracellular space. Different experimental proofs showed how their biochemical properties would fit to the idea of “diffusible” morphogens (5). Lipoprotein particles (6, 7), filopodia-surfing (8, 9) and transport on extracellular vesicles (10) were shown to confer biological Wnt activity in different systems [reviewed (11)]. “Extracellular vesicles (EVs)” is a term used for different sub-populations of membrane particles secreted from a plethora of cells into the extracellular space. Based on size and subcellular origin, they are discriminated into exosomes (50–100 nm), microvesicles (100–500 nm) and apoptotic bodies (>1000 nm) (12). Distinct proteins as well as lipid markers allow characterization of different types of EVs (13). CD9 or EMMPRIN are normally found in larger, plasma membrane-derived EVs, while components of the endosomal sorting complexes required for transport (ESCRT) machinery, such as Tsg101 and Alix are markers for the endosome-derived exosomes (14, 15). EVs are purified by differential ultracentrifugation, gel filtration or immunoprecipitation, while their size and composition are investigated by nanoparticles tracking, electron microscopy, immunoblotting and mass spectrometry. Standards for their purification and analysis have been defined and can help to increase reproducibility of EV studies (16). Currently, cell type-specific markers for EVs are missing.

Exosomes carrying Wnts were shown to play key roles under physiological conditions in different systems (17) (Table 1). The first evidence that Wnts might be released on membrane-bound structures came from studies of *Drosophila* Wnt, Wingless. In *Drosophila*, exosomes, containing the Wnt secretion factor Evi, transport Wnts across the Drosophila neural-muscular junctions (NMJ) and in the wing imaginal disc (10, 31, 32). Recently, a crosstalk was discovered in tooth development, activating Wnt/β-catenin signaling in mesenchymal cells via exosomal miRNA from epithelial cells (33).

**Table 1:** Recent studies about mechanisms and effects of Wnt release in mammalian systems

**Mechanism of Wnt release**	**Genes**	**Cell/Organism**	**Pathways/Details**	**References**
Paracrine exosomal Wnts	Wnt3a	Diffuse large B-cell lymphoma	Wnt/β-catenin signaling	(18)
Paracrine exosomal Wnts	Wnt2bPossibly also Wnt10a	Epidydemis/mouse	Differentiation/maturation Wnt/STOP	(19)
Paracrine exosomal Wnts	Wnt4	Human umbilical cord MSC in rat skin burn model	Angiogenesis and cell proliferation viaWnt/β-catenin signaling	(20–22)
Paracrine exosomal Wnts	Wnt4	Hypoxic colorectal cancer cells (HCT116, HT29)/endothelial cells (HUVEC)	HIFα-dependent Wnt4 expressionProliferation	(23)
Autocrine exosomal Wnts	Wnt11	Human umbilical cord MSC *in vitro*	Release stimulated by 3,3′-Diindolylmethane	(24)
Polarized exosomal Wnts	Apical/baso lateral Wnt3a,Apical Wnt11	Dog Kidney cells, MDCK	Basolateral Tsg101+Apical CD63 + apical Wnt secreted in a lipidation-independent manner	(25)
Paracrine exosomal Wnts	Wnt5b	Colon and pancreatic cancer cellsCaco-2, Panc-1	Several Wnts found in the supernatant after exosomes purification, such as Wnt3a and Wnt5a from L-cells	(26)
Paracrine exosomal Wnts	Wnt10b	Fibroblasts and breast cancer cells	Proliferation and migration	(27)
Crosstalk of Extravesicular Wnt	Wnt5a	Macrophages and breast cancer cells (SkBr3)	Wnt5a expression and cell invasion	(28)
Paracrine exosomes mobilize autocrine Wnts	Wnt11	Breast cancer cells (MDA-MB-231)	Cancer cell migration	(29)
Paracrine Exosome mobilize autocrine Wnts	Wnt10b	Cortical neuronsRat Optic nerve	Regeneration, mTOR	(30)
Neutral sphingomyelinases dependent trafficking of Wnts onto different EVs	Wnt3a and Wnt5a	Breast cancer cells (SkBr3)	Block of exosomes secretion increases microvesicles release	(13)
Paracrine Exosomal activating Wnt canonical	Wnt/β-catenin	Ischemia/reperfusion rat heart	Enhances cardiomyocyte survival and decreased apoptosis	(20)

As EVs are detectable in the circulation, it was conceived that their activity spreads systemically. Indeed, under pathological conditions, their functionality has long-range activity influencing metastatic niches far away from their source [reviewed in (34)]. Further examples are: (1) Tethering of autocrine Wnt11 to fibroblast-derived exosomes to influence the migratory phenotype of breast cancer cells (29); (2) colorectal tumor cells signal to endothelial cells (EC) by HIFα-induced exosomal Wnt4 secretion, activating Wnt/β-catenin signaling to increase migration and proliferation of ECs (23). Although this work is focused on cancer cells, activation of HIFα upon hypoxia plays a role in cardiac stress (23) and might have similar effects on EC crosstalk in cardiovascular pathologies.

## Implication and Caveats of the Wnt Signaling and EVs in the Heart

Heart function is based on a well-controlled communication system between different cell types. Although, EVs are well appreciated in the process of tumor and infection biology**,** research on cardiac EVs is increasing. So far no direct evidences for secretion of Wnt components on EVs from heart cells exist. Thus, we will integrate evidences from other fields, which may help to advances the knowledge on EVs/Wnt-mediated mechanisms in heart tissue.

### In Heart Development and Tissue Regeneration

Wnt signaling is crucial for embryonic development and tissue regeneration (3, 35). Specifically in cardiogenesis, activation of the Wnt/β-catenin signaling induces mesodermal formation, cardiac progenitor cell specification and maintenance, but inhibits further differentiation towards cardiomyocytes (36). Ectopic inactivation of the Wnt/β-catenin signaling in a tissue other than cardiac mesoderm, such as endoderm, is sufficient to trigger differentiation towards cardiac cells, indicating the central role of Wnt in cardiac cell formation (37). Several Wnt ligands are expressed in the early heart including Wnt2, Wnt2b, Wnt11, and Wnt8a, indicating the participation of canonical and non-canonical branches (38). Indeed, initial activation of Wnt/β-catenin signaling is followed by an activation of the Wnt/β-catenin-independent pathway, which represses the canonical signaling and regulates cell processes (39). Moreover, Wnt5a and Wnt11 promote cardiac differentiation in embryonic and adult stem cells through non-canonical pathways and may be necessary to balance β-catenin-dependent proliferation in the outflow tract (38, 40, 41). Hence, Wnt signaling is a network of inter-linked branches engaging different cell populations into intercellular crosstalk. Further details of the role of Wnt signals during cardiogenesis are extensively described elsewhere (36).

**Figure 1:**
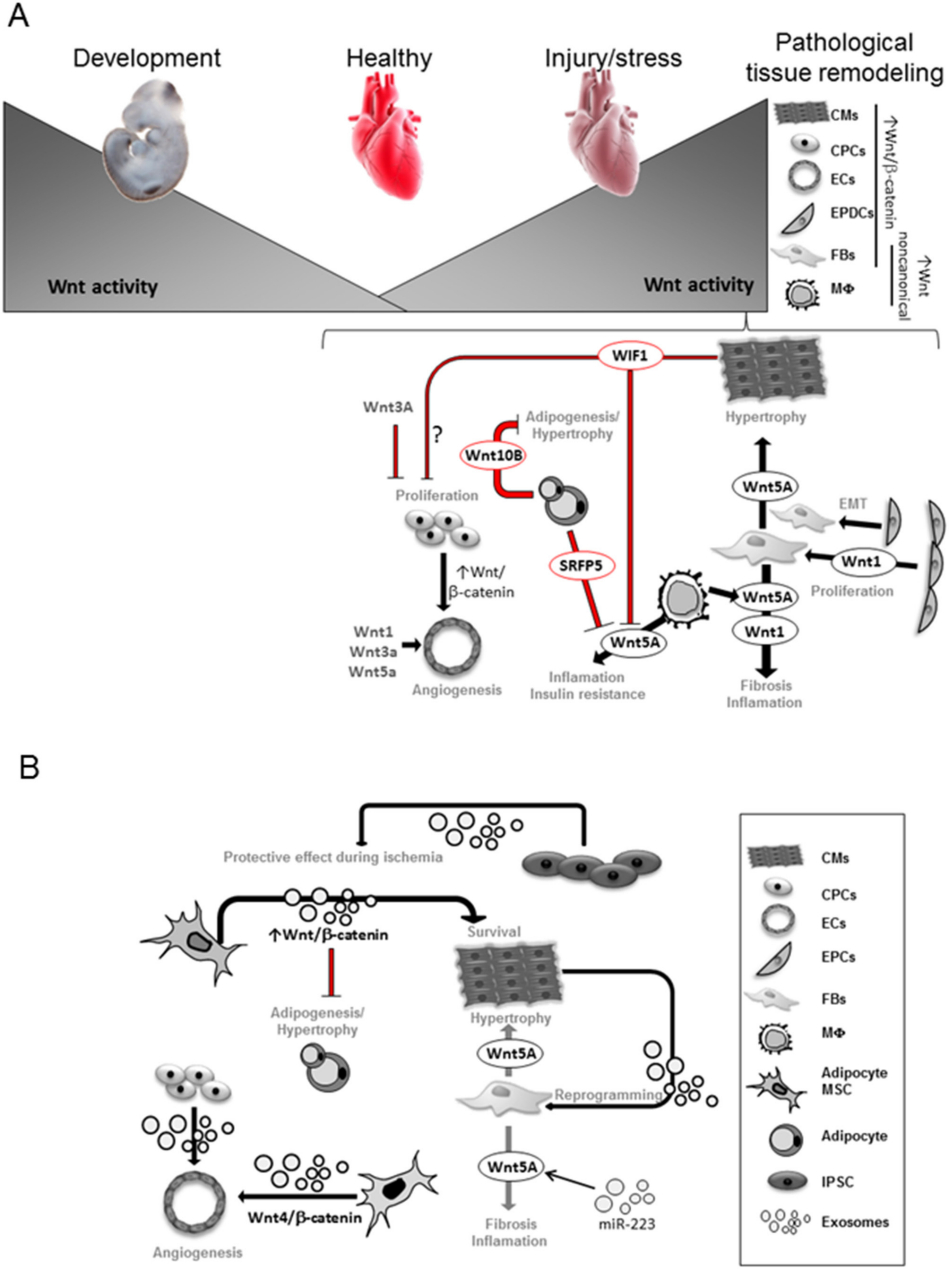
Wnt activity is necessary for heart development and becomes very low in the adult heart. Upon stress, Wnt signaling components are upregulated in different heart cells. Transcriptional dependent-canonical Wnt signaling is known to be activated in CPCs, epicardial-derived cells (EPDCs), fibroblasts (FBs), cardiomyocytes (CMs), endothelial (EC) and smooth muscle cells (35, 47, 48). Non-canonical Wnt components are mainly upregulated in Macrophages (MΦ) and FBs. Wnt1 secretion from EPDCs and FB promotes FB expansion and fibrosis (48); Wnt5a secretion from FB promotes CM-hypertrophy and fibrosis (49, 50); Wnt5a secretion from macrophages (MΦ) induces inflammation and insulin resistance leading to cardiovascular complications (49, 51). Secreted frizzled-related protein 5 (Sfrp5) by healthy adipocytes inhibits Wnt5a function from MΦ. The presence of unhealthy adipocytes with reduced Sfrp5 secretion increased Wnt5a activity (52, 53). Wnt10b from healthy adipocytes balances adipocytes growth (54). CMs-secreted Wnt Inhibitory Factor 1 (WIF1) reduces Wnt5a activity and may affect CPCs proliferation (55). Secreted Wnt1, Wnt3a, Wnt5a as well as activation of Wnt/β-catenin induced by CPCs stimulate angiogenesis (48). Exosomes derived from cardiomyocytes (CMs) showed ability to reprogram fibroblasts (FBs) *in vitro* (56, 57). MiR-233 upregulates Wnt5a expression and miR-223 can be found in exosomes (58), thus Wnt5a regulation in cardiac failure may involve exosomal trafficking. Exosomes derived from adipocyte-derived mesenchymal stem cells (MSCs) was shown to activate Wnt/β-catenin signaling pathway, which may affect CM survival and constrains adipogenesis (20). Exosomes derived from umbilical cord MSCs showed a pro-angiogenic effect by delivering Wnt4 and activating Wnt/β-catenin signaling in endothelial cells (ECs) (21). Exosomes secreted from human induced pluripotent cells (iPSCs) showed protective effects on ischemic myocardium (59).

Notably, heart regeneration mechanisms vary among species. In contrast to the limited injury-induced regeneration in early stages of life in adult mammal hearts, lower vertebrate like amphibian and teleost fish have sufficient regenerative capacity upon injury mainly by dedifferentiation of cardiomyocytes (35). In mouse, Wnt signaling exerts a similar role on adult cardiac progenitor cell (CPC) homoeostasis as observed during embryogenesis. Wnt/β-catenin activation impairs cardiomyocytes lineage differentiation and enhances endothelial cell (ECs) expansion, whereas its inactivation increases cardiomyocytes and reduced EC lineages (42–44). Accordingly, intra-myocardial injection of Wnt3a post-ischemia reduces CPC differentiation into cardiomyocytes (45). However, the role of Wnt signaling and most importantly the intercellular crosstalk in endogenous regeneration remains unclear. Interestingly, in the regenerative zebrafish hearts Wnt/β-catenin pathway is reactivated upon injury (35). In a recent study, one-day postnatal murine cardiomyocytes, with high regenerative potential, showed enriched Wnt signaling gene networks after ischemic injury (46). Since Wnt signaling becomes inactivated in the postnatal heart during later stages, it was speculated that reactivation of the signaling will confer regenerative capacity to the adult heart, however with impaired cardiac performance. This may imply an initial protective mechanism of the stressed heart to preserve cardiomyocytes function, which eventually fails upon sustained activation of the pathway (Figure 1), probably due to a low developmentally permissive transcriptional state of the adult cardiomyocytes (46).

### The Stressed Heart: Specific Wnt Component Regulation

Lack of robust regenerative response, upon stress/injury in the adult mammalian heart, results in adaptive tissue remodeling to sustain cardiac output. This finally leads to heart failure development characterized by a switch towards fetal metabolism and re-expression/elevation of developmental genes (60), including genes of the Wnt signaling pathway (13). Therefore, Wnt/β-catenin signaling has been considered a potentially therapeutic target for heart disease (35, 61–64). In the healthy adult heart, Wnt signaling is quiescent but becomes reactivated in different cell types in the ischemic and hypertrophic heart (Figure 1) (62–65). Specifically, Wnt/β-catenin activation is found in epicardium, fibroblasts, ECs, smooth muscle cells and CPCs (35) and in cardiomyocytes of the human failing heart (47). Conversely, Wnt inhibition appears to protect the heart from pathological ventricular remodeling (61, 63, 66, 67).

Recent studies indicate that the exosomal content is highly regulated in the heart by various stress conditions and that cardiomyocytes and cardiac fibroblasts release exosomes in *in vitro* studies (56, 68, 69). Moreover, Wnt ligands, FZDs and SFRPs are elevated after ischemic heart injury (48). These observations allow speculating that Wnts traveling on exosomes upon cardiac remodeling may be part of the maladaptive response. After myocardial infarction (MI), cardiac fibroblasts respond to Wnt1 in an autocrine fashion to induce proliferation and fibrogenic genes expression (48). Wnt1, Wnt3a, and Wnt5a regulate proliferation and migration of ECs. Moreover, after MI, β-catenin accumulates in ECs of the rat heart, which suggests activation of canonical Wnt signaling (48). Accordingly, antagonizing Wnt3a/Wnt5a binding to its receptors FZDs prevents heart failure upon ischemia (61). Wnt3a and Wnt5a were already found in exosomes (10, 26, 28). Interestingly, cell-autonomous regulation of Wnt signaling by enhancing β-catenin export on exosomes and reducing its cellular pool was described in tumor cells (70). An exciting idea will be to stimulate exosomal export of β-catenin, thereby reducing signaling activity in heart remodeling and preventing heart failure development.

Upon MI, macrophages are a source of non-canonical Wnts. Genetic ablation of Wnt signaling in mice results in macrophages with anti-inflammatory, reparative, and angiogenic properties and improved left ventricular function and remodeling after MI, possibly by the elimination of non-canonical signaling (51). In the failing myocardium, Wnt5a was most prominently upregulated in cardiac fibroblasts and elevated circulating Wnt5a levels were associated with adverse outcomes in patients with dilated cardiomyopathies (49). In mouse and human cardiac fibroblasts, recombinant Wnt5a upregulated the release of Interleukine (IL)-6 and Tissue Inhibitor Of Metalloproteinases 1 (TIMP-1). This might promote myocardial inflammation and fibrosis contributing to heart failure progression (50). Moreover, Wnt5a is known to stimulate hypertrophy in cultured cardiomyocytes (49). Increasing evidence suggests that miR-223 upregulates Wnt5a expression (50) and miR-223 can be found in exosomes (58), hence Wnt5a regulation in cardiac failure may involve exosomal trafficking.

Upon MI, Secreted frizzled-related protein 5 (Sfrp5) functions as an extracellular inhibitor of non-canonical Wnt signaling (52) that antagonizes the pro-inflammatory activity of Wnt5a. Sfrp5 is highly expressed by healthy adipocytes, thus may act as a paracrine cardio-protective adipokine. Obese people with “unhealthy adipocytes” with reduced expression of Sfrp5 and high Wnt5a have an associated insulin resistance with a high risk of cardiovascular complications (53). Moreover, Wnt5a overexpression in myeloid cells augments adipose tissue inflammation; promotes pro-inflammatory cytokines by macrophages and impairs glucose homeostasis (54). Accordingly, Wnt5a ablation in obese mice ameliorates insulin resistance. Thus, Wnt5a crucially mediates cellular crosstalk to finally affect glucose metabolism and cardiac homeostasis. Additionally, Wnt5a induced hypertrophic NFAT activation in cardiomyocytes *in vitro* (49). Another Wnt, Wnt10b constrains mouse white adipose tissue expansion by inhibiting pre-adipocyte differentiation, modifying adipokine secretion and immune-modulatory roles of fat tissue (54). Of note, adipose tissue is an important source of circulating exosomal miRNAs in mice and humans and may regulate whole-body metabolism (71). Exosomes derived from adipocyte-derived mesenchymal stem cells (ADMSCs-ex) significantly ameliorated ischemia/reperfusion-induced myocardial necrosis and apoptosis in rat heart (20). The mechanisms underlying the cardioprotective effects of ADMSCs-ex may be associated with activation of Wnt/β-catenin signaling, a critical regulator of survival and apoptosis of cardiomyocytes (Figure 1B).

Hypoxic cardiomyocytes upregulate Wnt Inhibitory Factor 1 (WIF1) (72), which interferes with non-canonical Wnt signaling in monocytes and macrophages and reduces pro-inflammatory activation upon ischemia. In patients with hypoxia-associated disorders such as MI, stroke and pre-eclampsia, an increase of circulating EVs indicates a role of EVs as biomarkers in these pathophysiological states (55). EVs could be regarded as radar signals that confer a population overall fitness and unify their individual regulatory patterns.

Taken together, Wnt signaling activation is key in pathological heart remodeling and EV-mediated signaling may participate in this activation. The contribution of EV-mediated Wnt signaling to block tissue regeneration needs further investigation in order to engineer EV-modifications allowing the recovery of developmental plasticity.

## EV-Signaling in Cardiovascular Cells

Proper cardiac function relies on communication of cardiomyocytes with other cell types including smooth muscle cells, EC, fibroblasts and immune cells (73). These cells function together by interacting physically or via secreted factors, including lipids, peptide, nucleotides and miRNAs. The adult myocardium secretes exosomes to mediate cell-cell communication (74). Upon cardiac stress, fibroblast-secreted exosomes enriched in miR-21*, which is normally degraded, is taken up by cardiomyocytes to induce cell hypertrophy (69). Moreover, during MI, distinct exosome-contents from border zone and healthy heart cells suggest an adaptive response to injury defined by exosome secretion (75). Primary cardiomyocytes were capable of secreting EVs with the ability to reprogram fibroblasts *in vitro* (56, 57). Thus, cardiomyocytes are able to transfer signals to direct neighboring cell fate (Figure 1B). Exosomes are not only potential circulating biomarkers (76) but they are also considered for their potential therapeutic anti-fibrotic and angiogenetic effects as antioxidants protecting cardiomyocytes (77).

### Exosome-Mediated Signaling in Endogenous Progenitors

Current data indicate a role for Wnt signaling in the homeostasis of CPCs in the adult heart. However, cardiac functionality may not be directly affected by changing the balance of this small pool but by secreted products (78). In line with this, stem cell injections in the adult heart were suggested to mediate a paracrine regeneration through secreted signals (72) and CPCs are a source of exosomes (79). Under hypoxia conditions, these cells secrete “pro-regenerative” exosomes inducing proliferation of ECs (73). Cardiomyocyte progenitor and mesenchymal stem cell-exosomes have powerful pro-angiogenic effects (80) (Figure 1B). Given, the above-discussed action of Wnt activation on stimulating ECs fate, it is tempting to speculate that those “endothelial-pro-regenerative” exosomes may signal through the Wnt pathway.

Since exosomes are carriers of both protective and pathological signals, a better understanding of their content and effect on recipient cell will help to define therapeutic utilities of EVs. And will broaden our understanding of how cells and organs communicate among each other (73).

### Regenerative Potential of EVs

Tissue repair requires not only the presence of cells capable to restore damage tissue, but more importantly, requires a microenvironment promoting tissue regeneration. A recent study showed that fibroblast-derived exosomes relocalize Wnt10b into lipid rafts, activating mTOR and promoting axonal regeneration in an inhibitory environment after optic nerve injury (30). It seems that Wnts on different EVs have similar signaling capacities and that loading with specific content is more relevant for their functionality than the EVs used to mobilize (28). This is in agreement with biotechnological approaches where liposomal packaging of Wnts confers a longer stability and high signaling capacity in regeneration models (81, 82). This microenvironment can be created by exosomes with defined contents, ideally delivering signals affecting cell recruitment, differentiation and immunomodulation. Given the important role of exosomes in tissue regeneration in pre-clinical models, further studies addressing the EVs-mediated signaling are of high interest. Elucidating these mechanisms will offer a great platform for EVs engineering for personalized medicine.

Human pluripotent stem cells (hPSCs) and induced pluripotent cells (iPSCs) have been widely used in translational medicine for their enormous therapeutic potential in tissue repair and regeneration. Isolated exosomes secreted from iPSCs showed protective effects on ischemic myocardium by transferring the endogenous molecules to salvage the injured neighboring cells (59). In this regard, iPSCs-derived exosomes could be used for clinical application as autologous bioactive, cardio-protective exosomes to treat heart diseases and become a clinical tool for personalized medicine (75). Exosomes derived from umbilical cord mesenchymal stem cells showed a pro-angiogenic effect by delivering Wnt4 and activating Wnt/β-catenin signaling in ECs (21). Since activation of Wnt/β-catenin signaling is also pro-angiogenic in the adult heart, it is tempting to speculate that endogenous CPCs may also use EVs for pro-angiogenic signaling. Activation of canonical Wnt signaling was also reported in osteoblast-derived exosomes carrying miRNA to promote osteogenic differentiation. Thus, not only Wnt components may be carried onto EVs but also miRNA regulating Wnt signaling may be involved in cell-cell communication.

Cell therapies can directly support regenerative processes by forming new functional tissues or supporting tissue generation via paracrine mechanisms. Dissecting the precise role of Wnt signaling in cardiac tissue regeneration and the potential use of synthetic EVs may help tailor therapeutic approaches aiming to restore tissue functionality in a non-regenerative environment such as the heart. Moreover, human PSCs provide an excellent tool to address EV-mediated signaling in the context of early cardiogenesis. Developing protocols for exosomes isolation in their* in vivo* environment will allow cell-type and cargo-specific EVs and will enormously advance the field of EV-mediated signaling.

## Conclusion

Modulation of Wnt signaling is crucial for tissue homeostasis in the developing and postnatal heart. However, the role of Wnt/β-catenin-dependent and -independent pathways in the intercellular crosstalk of heart cells is not fully understood. Activating or inactivating branches of the Wnt-network in specific target cells may be attractive to modulate pathological processes in the cardiovascular system or to enhance regenerative capacities of stem cell therapies. Many of these mechanisms might be mediated by EVs. Hence, understanding Wnt signal transduction via EVs between cell populations and tissues will advance our strategies for therapeutic modulation of these pathways.

## Author Contributions

Both authors conceived, discussed and wrote the review.

## Conflict of Interest Statement

The authors declare that the research was conducted in the absence of any commercial or financial relationships that could be construed as a potential conflict of interest.

The reviewer PY and handling Editor declared their shared affiliation.
